# An epidemiological analysis of acute flaccid paralysis and its surveillance system in Iraq, 1997-2011

**DOI:** 10.1186/1471-2334-14-448

**Published:** 2014-08-20

**Authors:** Jagar A Jasem, Kawa Marof, Adnan Nawar, Yosra Khalaf, Faisal Al-Hamdani, Sagvan Ali, Andre C Kalil, KM Monirul Islam

**Affiliations:** School of Medicine, Faculty of Medical Sciences, University of Duhok, Duhok, Kurdistan Region Iraq; Internal Medicine Department, Ohio State University, Columbus, Ohio USA; Directorate of Preventive Health Affairs, Directorate General of Health, Duhok, Kurdistan Region Iraq; National Communicable Disease Control, Ministry of Health, Baghdad, Iraq; AFP Surveillance, Ministry of Health, Baghdad, Iraq; National Polio Laboratory, Ministry of Health, Baghdad, Iraq; Division of Infectious Diseases, Department of Internal Medicine, University of Nebraska Medical Center, Omaha, Nebraska USA; College of Public Health, University of Nebraska Medical Center, Omaha, Nebraska USA

## Abstract

**Background:**

Acute flaccid paralysis surveillance (AFP) is an essential strategy of the WHO’s Polio Eradication Initiative. This is the first study conducted to estimate the incidence, etiology, distribution, and surveillance performance of AFP in Iraq.

**Methods:**

Surveillance data about the AFP cases under the age of 15 years reported from Iraq during January 1997 to December 2011 were depended in the current study.

**Results:**

A total of 4974 cases of AFP were reported from Iraq during the study period, with an annual incidence of 2.5/100,000 population. Guillain-Barré syndrome represented more than half of the reported cases (N = 2611, 52.5%), followed by traumatic neuritis (N = 715, 14.4%), and other CNS infections (N = 292, 5.9%). Poliomyelitis accounted for 166 (3.3%) of cases, the last reported case being in January 2000. Surveillance performance showed that all, but two, indicators were below the required WHO recommended levels.

**Conclusions:**

AFP surveillance remains the gold standard method for poliomyelitis detection. It witnessed dramatic changes over the last two decades. This has raised people’s and clinicians’ awareness to the importance of promptness in notifying suspected cases and timely transportation of stool specimens to the National Poliovirus Laboratory in Baghdad, or alternatively having more than one laboratory for poliovirus detection in the country, all of which are very useful measures to increase the surveillance performance in the country.

**Electronic supplementary material:**

The online version of this article (doi:10.1186/1471-2334-14-448) contains supplementary material, which is available to authorized users.

## Background

Poliomyelitis, or polio, is a highly contagious viral infection caused by poliovirus. Like most other *Enterovirus* RNA viruses, poliovirus is transmitted by the feco-oral route. The vast majority of infections are subclinical (>90%), while the remainder present with a self-limited illness of fever, myalgia, malaise, sore throat and headache (abortive polio) or aseptic meningitis (i.e. non-paralytic polio). The paralytic disease is by far the least common presentation, accounting for 1 per 1000 cases among infants and 1 per 100 cases among adolescents [1, 2].

In 1988, the World Health Organization (WHO) adopted the Global Polio Eradication Initiative (GPEI), aiming to eradicate polio by the year 2000 [3]. Although polio outbreaks continue to occur, polio cases have witnessed a dramatic decrease by >99.9% since 1988 [4]. Along with attaining high routine vaccination coverage rate, conducting National Immunization Days (NIDs), and adopting “mopping-up” immunization; acute flaccid paralysis (AFP) surveillance is an essential strategy of the GPEI. It aims to identify high risk areas or groups, monitor progress of polio status, indicate the need for supplementary strategies if widespread outbreaks occur or “mopping-up” immunization if restricted to limited foci, as well as to certify a country polio-free. The latter requires that “there are no reports of new cases of poliomyelitis caused by wild poliovirus for three successive years”. It also requires evidence that a country can detect a case of paralytic polio should it occur [5].

Iraq is a Middle Eastern country located in western Asia covering an area of 435,052 km2 with 18 governorates (provinces). It is bordered to the north by Turkey, to the east by Iran, to the west by Syria and Jordan, and to the south by Saudi Arabia and Kuwait. The population of Iraq grew from about 22,046,000 in 1997 to about 33,330,000 in 2009 [6]. Immunization against polio was mandated by the Iraqi Ministry of Health in 1985, according to which each child should receive a first dose of OPV within 2 weeks from birth. Subsequent doses should be administrated at 2, 4 and 6 months. National immunization days (NIDs) have been at least biannually launched in Iraq since 1995. National reporting started in Iraq since 1997. Virologic classification was introduced in 2000. Iraq has achieved and maintained these certification standards since 1997 [7].

Many studies have been conducted on the causes of AFP and its surveillance system performance, including recent studies from different countries all around the world [8–12]. However, unfortunately, no studies have been carried out with the aim to estimate the incidence, etiology, distribution, and surveillance performance of AFP in Iraq.

## Methods

### Study design and population

The surveillance database of AFP cases under the age of 15 reported from Iraq during January 1997 to December 2011 was depended in the current study. The study was approved by the Ethical Committee of the Faculty of Medical Sciences/University of Duhok, Duhok, Iraq and the Institutional Review Board (IRB) of the University of Nebraska Medical Center, Omaha, Nebraska, USA.

### AFP surveillance organization in Iraq

AFP information is routinely included in the weekly and monthly reporting system from the Preventive Health Department (PHD) in the Directorate General of Health (DGoH) of every Iraqi province, even if there has been no reported case (routine surveillance “zero-reporting”). Notifications are done via mailing the standard communicable notification forms designed by the Ministry of Health (MOH) to the Communicable Disease Control Unit (CDCU) of the regional PHD. Active surveillance for the suspected cases of AFP is ideally done within 48 hours via a designed investigator from the CDCU visiting the reporting sources (hospitals, rehabilitation centers, or private clinics). Case investigation is done using a WHO-standardized form.

According to the WHO recommendations, two stool specimens are collected from each suspected case with an interval of 24-48 hours between collections, given that no more than two months have elapsed since the onset of paralysis [5]. Moreover, one stool sample is collected from at least 3 contacts less than 15 years of age who have been in direct contact with the index AFP case within one week prior to the onset of paralysis and/or within two weeks after onset. If the contact numbers are inadequate, sampling from children in the neighbourhood are collected. Following collection, the specimens are kept in a cold box between frozen ice packs at 4-8°C to be sent to the National Polio Laboratory in Baghdad accompanied by a WHO-standardized form for laboratory analysis.

At least 60 days after the onset of paralysis, all surviving patients are re-examined by an Expert Committee member for residual paralysis in the premise that such a finding would provide further evidence that the cause of paralysis is poliovirus.

### Case definition of AFP

A “suspected polio” case is defined as “acute, flaccid paralysis in a child aged <15 years including Guillain-Barre syndrome; or any paralytic illness in a person of any age when polio is suspected [5]”.

“Suspected polio” or AFP is a temporary diagnosis that needs to be finally classified by an Expert Committee into either: confirmed polio, polio-compatible, or discarded. Such a classification is done on either clinical or virological basis; the latter being used in Iraq since the year 2000 [5].

### Surveillance performance indicators

The performance indicators were evaluated in comparison to specific WHO recommended cut-off levels described in the Results and Discussion section [5].

## Results and discussion

### Epidemiology of AFP

Given the heterogeneity of AFP etiology, each disease has its own epidemiological characteristics. Nevertheless, these characteristics are largely driven by GBS epidemiology as it represents the vast majority of reported cases (see below).

**Figure 1:**
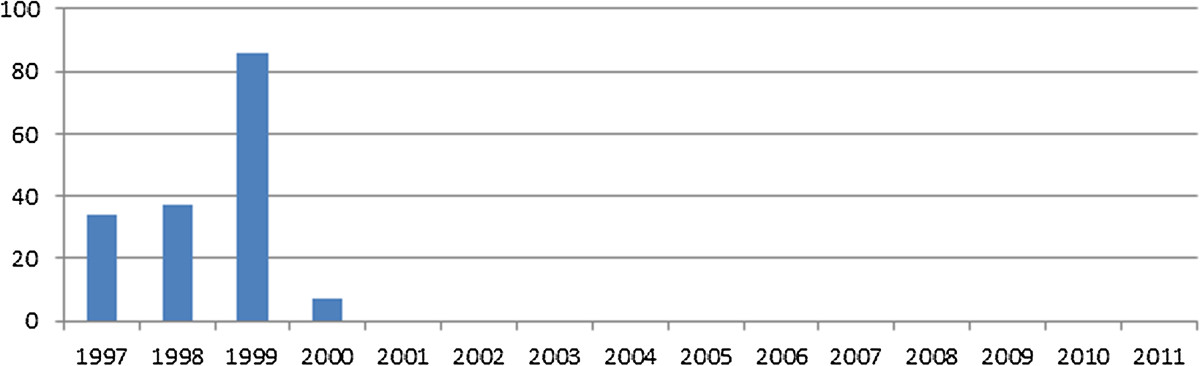
**Number of poliomyelitis cases per year, Iraq, 1997-2011.**

**Figure 2:**
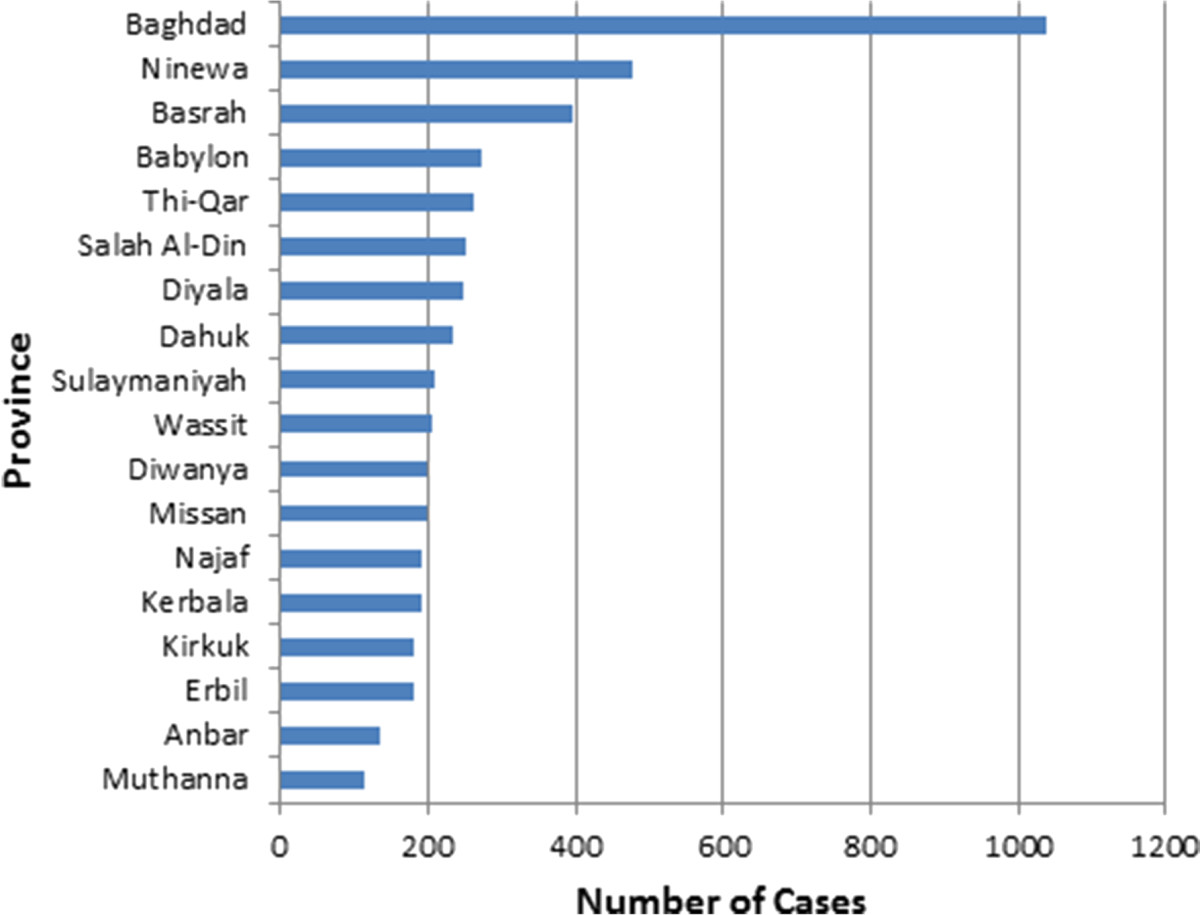
**Number of AFP cases reported per province, Iraq, 1997-2011.**

**Figure 3:**
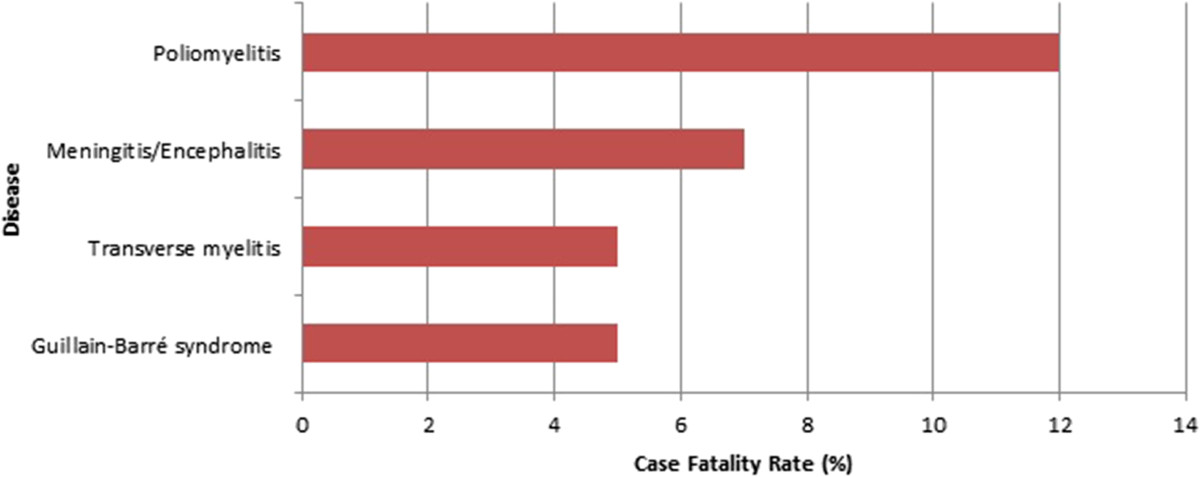
**Case fatality rate of selected causes of AFP, Iraq, 1997-2011.**

A total of 4974 cases of AFP were reported from Iraq between January 1997 and December 2011. Of those, 2848 (57.3%) were males and 1990 (40%) were females. The gender of the remaining 136 (2.7%) was unidentified in the database. The age of reported cases ranged from 5 days to 14 years, with a mean of 3 years and median of 2.5 years. The annual incidence of AFP was calculated to be 2.5/100,000 population from 1997-2011. Guillain-Barré syndrome represented more than half of the reported cases (N = 2611, 52.5%), followed by traumatic neuritis (N = 715, 14.4%), and other CNS infections (5.9%) (Table 1). Poliomyelitis accounted for 166 (3.3%) of cases, the last reported case being in January 2000 (Figure 1). Other conditions including brain tumors, intracranial hemorrhage, electrolyte imbalance, epilepsy, hypokalemia, and myopathy accounted for 622 (12.5%) of cases. The exact cause of AFP was not identified in 568 (11.4%) of reported cases. About 96% of cases were reported from the public hospitals. All cases were of Iraqi nationality. The greatest number of cases was reported from the provinces of Baghdad, Ninewa, and Basrah (Figure 2).Case-fatality rate of poliomyelitis was the highest among all other causes of AFP (Figure 3).

**Table 1:** **Causes of AFP in Iraq, 1997-2011**

Cause	Number (%)
Guillain-Barré syndrome	2611 (52.5)
Traumatic neuritis	715 (14.4)
Meningitis/Encephalitis	292 (5.9)
Poliomyelitis	166 (3.3)
Myopathy	89 (1.8)
Hypokalemia	74 (1.5)
Unknown	568 (11.4)
Others	459 (9.4)
**Total**	**4974 (100)**

### AFP case classification

**Figure 4:**
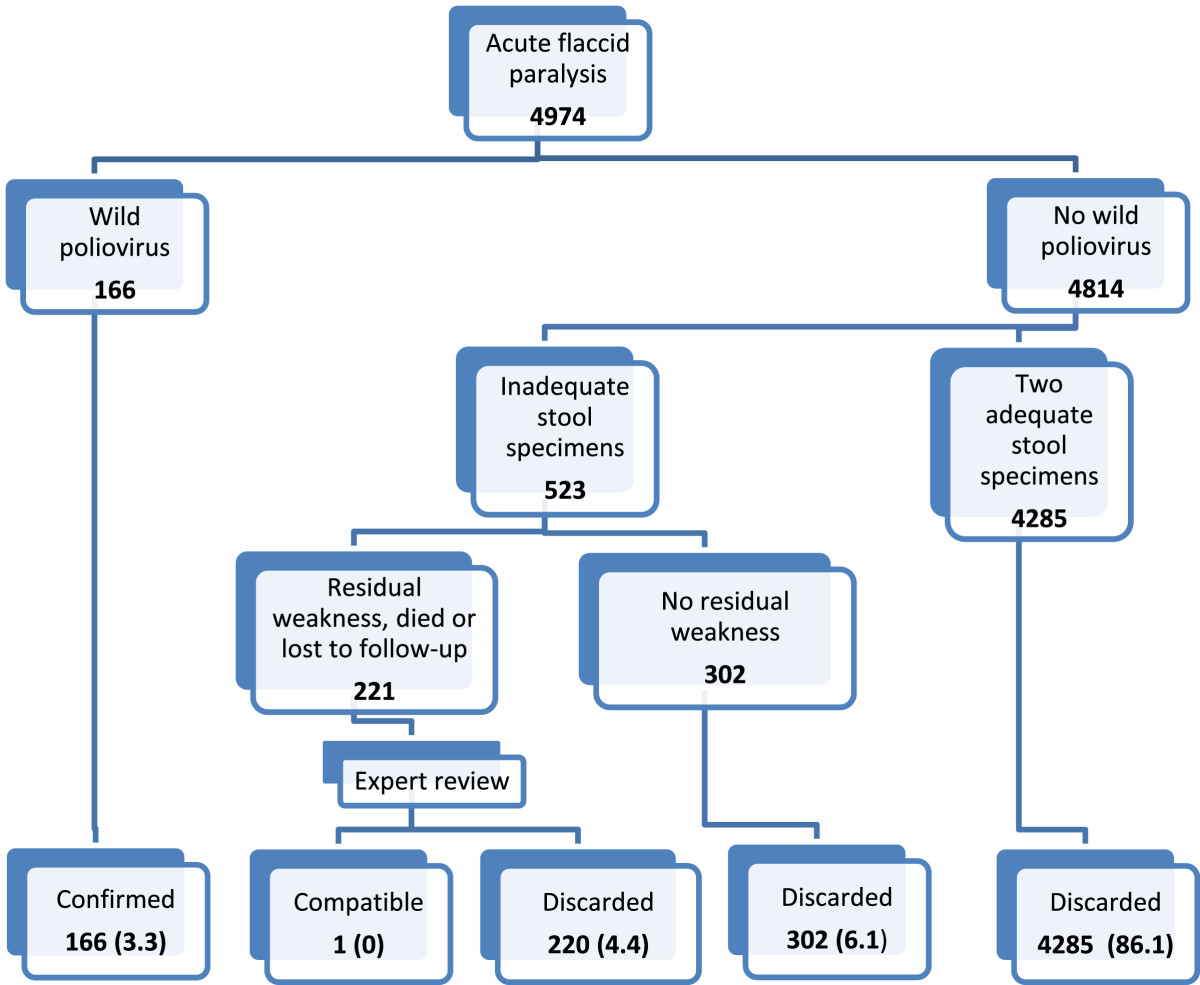
**Virological flowchart and case classification of the reported AFP cases, Iraq, 1997-2011.**

Wild polio virus was detected in 166 samples. In the remainder, the stool specimens were adequate, and hence sufficient to exclude poliomyelitis, in 4285 (86.1%) of cases (Figure 4). Of the remaining 523 cases with inadequate stool specimens, paralytic poliomyelitis was excluded by resolution of paralysis when followed after 60 days of the onset of paralysis in 302 cases, while the Expert Committee excluded poliomyelitis in 221 cases with residual paralysis, lost to follow-up, or died before the expected follow-up date. One case of those who died before the expected follow-up date was labelled as “compatible” with poliomyelitis. The case was reported in August 2000.

### Impact of political and socio-economic changes on polio eradication strategies in Iraq

Polio eradication strategies in Iraq witnessed dynamic changes over the last two decades. Mandating routine immunization against polio was followed by significant increase in coverage rates in late 1980s [7]. However, following the 1990-1991 Gulf War and the subsequent economic embargo, public health budget sustained a serious decline by 90% [13]. Polio, like many other vaccine-preventable diseases, was widely reported in Iraq during early 1990s due to lack of vaccine supply, poor maintenance of vaccine cold chain, and disrupted infrastructure of the health care system in the country. Following the Oil for Food Program adoption by the UN Security Council in 1995, at least two polio vaccination campaigns were held in Iraq every year with resultant increase in coverage rates to those of pre-1990s era [14].

As an emergent response to the polio outbreak in 1999, Iraqi Ministry of Health with the assistance of WHO and UNICEF, conducted two rounds of eminently successful vaccinations of eligible population through National Immunization Days (NIDs) in October and November 1999 [5].

Following the 2003 war, more than 30% of primary care clinics, 12% of hospitals and 15% of child care clinics were looted or damaged. Likewise, the two central public health laboratories were destroyed and the Institute of Vaccine and Sera was stripped of equipment and vaccine supply [14]. Immunization campaigns were successfully re-launched by the Disaster Assistance Response Team represented by UNICEF and WHO in the second half of 2003. However, Polio vaccine efforts were significantly hindered by shortage of vaccine supply, failure of cold chain maintenance, and the lack of security and sectarian violence in the Southern and Central provinces [13–16]. On the other hand, the semi-autonomous northern Kurdish provinces witnessed significantly better primary health care services owing to the political and economic stability in the region. Neonatal polio vaccine coverage rate exceeded 70% in Kurdistan Region by 2010 compared to an opposite trend to 40% in the central and southern provinces [14].

A recent study has demonstrated that children exposed to the war were over 20 percentage points less likely to be immunized compared with non-exposed ones, even after controlling for such confounders as individual and household characteristics, year of birth and province of residence [14].

### AFP surveillance evaluation

**Figure 5:**
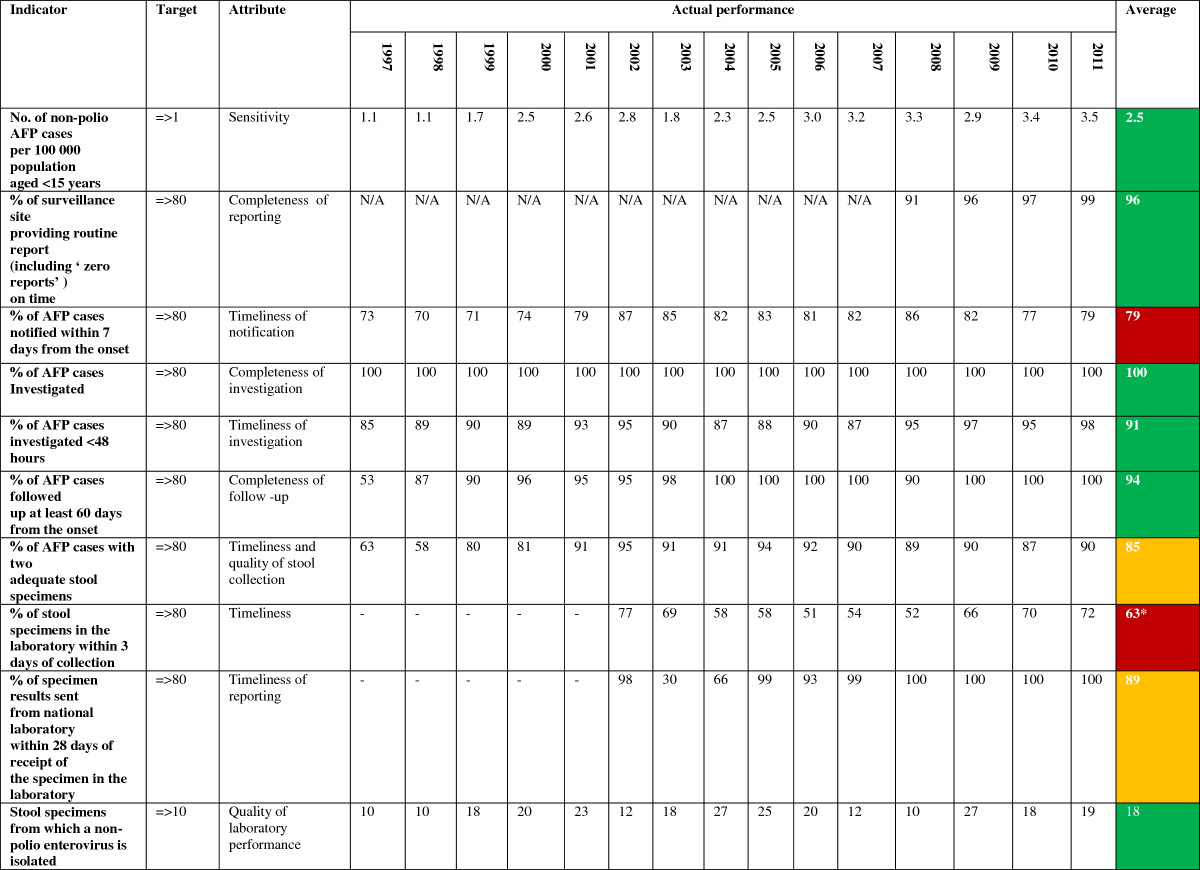
**AFP surveillance performance according to pre-selected WHO indicators, Iraq, 1997-2011.**

Evaluation of the AFP surveillance system is based on a number of pre-set WHO indicators (Figure 5), including:

#### Sensitivity and positive predictive value

Sensitivity refers to “the proportion of cases of a disease (or other health-related event) detected by the surveillance system [17]”. The annual detection of at least one non-poliomyelitis AFP case per 100,000 population under the age of 15 years is the WHO’s recommended sensitivity measure for the AFP surveillance system. There was a notable increase in the reported annual rates of AFP in Iraq from 1.1 cases/100,000 population under the age of 15 years in 1997 to 3.5 cases/100,000 population under the age of 15 years in 2011. The national average rate from 1997-2011 was 2.5 AFP cases/100,000 population under the age of 15 years.

However, a better way to estimate the actual incidence of AFP in the population is to use the capture-recapture method for the cases in which the national-registered cases are compared to the hospital-registered ones as primary and secondary case ascertainment sources, respectively [18–20]. Obviously, this is a laborious method and difficult to apply to the whole country as it is an exceptionally hard task to evaluate all hospital records for AFP cases, especially since Iraq does not currently have reliable systems for retrospective patient data review [13, 19, 21].

The positive predictive value, defined as “the proportion of reported cases that actually have the health-related event under surveillance [17]”, is difficult to determine for the AFP surveillance system in Iraq as the diagnosis is strictly clinical and only the true AFP cases are virtually registered in the local and national databases.

#### Completeness

Completeness of reports is an important quantitative measure of the “acceptability” of public health surveillance systems, the latter reflecting “the willingness of persons and organizations to participate in the surveillance system [17]”. The following completeness measures are recommended for AFP surveillance:

- Completeness of reporting: Almost 96% of expected routine (weekly or monthly) AFP surveillance reports from all over Iraq were received on time, including zero reports.
- Completeness of investigation: Clinical and virological investigation was done for all AFP cases having ‘adequate’ stool specimens collected.
- Completeness of follow-up: A follow-up examination for residual paralysis was performed for 94% of AFP cases at 60 days after the onset of paralysis.

#### Timeliness

**Figure 6:**
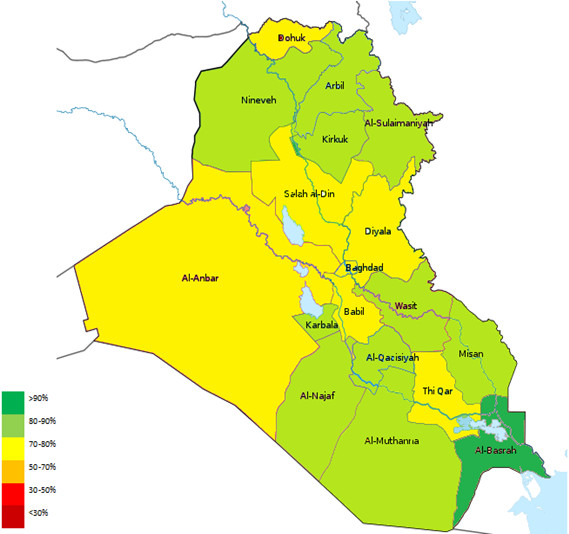
**Map of Iraq depicting the percentage of AFP cases notified within 7 days from the onset of paralysis, Iraq, 1997-2011.**

**Figure 7:**
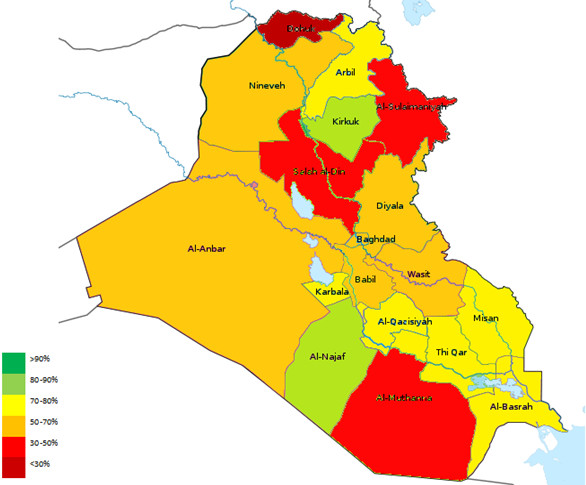
**Map of Iraq depicting the percentage of stool Specimens received by the laboratory within 3 days of collection, Iraq, 1997-2011.**

Another quantitative measure of surveillance program “acceptability”, timeliness refers to “the speed between steps in a public health surveillance system [17]”. In case of AFP surveillance, the following intervals are measured:

- Timely notification: This refers to the interval between the onset of paralysis and the notification of the AFP Surveillance Team at the local Directorate of Health, which is estimated to be 7 days. This is a crucial step to summon public health effort needed to prevent further transmission of infection. Unfortunately, this indicator is slightly under the cut-off level of 80% in Iraq. This might reflect either a delay in seeking treatment or delayed notification by the caregiving physicians (Figure 6).
- Timely investigation: Refers to the interval between case notification and investigation by the designated AFP Surveillance Team. It reflects the response of the AFP Surveillance Team to health-related emergencies. The vast majority of reported AFP cases (91%) in Iraq were investigated within the allocated 48-hour interval.
- Timeliness of stool collection: There was a steady increase in the proportion of cases from which two adequate stool specimens (>8 grams) were collected with an interval of 24-48 hours between collections, given that the onset of paralysis was within 2 months of notification. This reflects better awareness of patients’ families and medical caregivers to the importance of early detection and technique of stool collection.Timeliness of specimen receipt by the laboratory: Unfortunately, stool cultures from only 63% of cases were received by the National Poliovirus Laboratory in Baghdad within 3 days of collection. Only 2 of the 18 Iraqi provinces maintained a level above the cut-off point of 80% (Figure 7). This is mainly due to difficulty in inter-and-intra-provincial transportation, which is largely attributed the challenging security situation in the country.
- Timeliness of result reporting: Prompt analysis and feedback from the National Poliovirus Laboratory in Baghdad to the local and international health authorities in cases of emergency is essential to cut the chain of outbreak transmission. Results of virological stool studies for 89% of cases were appropriately reported within 28 days of receiving the cultures.

#### Quality of laboratory performance

The Iraqi National Poliovirus Laboratory in Baghdad is a WHO-accredited laboratory within the Eastern Mediterranean Region (EMR) Poliovirus Laboratories network [7]. The sensitivity of the laboratory to detect poliomyelitis is reflected by its ability to detect non-poliomyelitis enteroviruses (NPEV) in at least 10% of cases, given that NPEVs have a worldwide distribution and they are not targeted for eradication [22]. Besides the timely processing of stool culture analysis and reporting of results, the National Poliovirus Laboratory in Baghdad was able to detect non-poliomyelitis NPEVs in 18% of cases.

#### Usefulness

The usefulness of a public health surveillance system is indicated by its ability to fulfil its objectives related to disease prevention, health promotion, and policy decision-making [17]. Since its adoption in 1997, the AFP surveillance system in Iraq has proven its efficacy in detecting AFP cases and successfully met most of the timeliness and completeness parameters of surveillance. Via documentation of the causes of AFP in the country, the AFP surveillance system resulted in the certification of Iraq as polio-free. The AFP surveillance system provides important estimates of morbidity and mortality related to various causes of paralysis among Iraqi children and stimulates research about potentially effective control and policy-making measures.

#### Representativeness

The representativeness of a public health surveillance system is reflected in its ability to document the time-trend for disease occurrence and its geographic and demographic distribution [17]. The AFP surveillance system in Iraq covers the age groups below 15 in all of the 18 provinces in the country, regardless of their gender, socioeconomic status, and residence within or outside the capital city of the province. The vast majority of the cases (96%) were reported from public hospitals that provide universal health care over all the country. However, there remains a chance of underreporting from the private clinics that can function remotely from the direct supervision of public health authorities.

#### Cost-effectiveness

Since its adoption by WHO in 1988, the GPEI has successfully prevented more than 8 million life-long paralytic cases of poliomyelitis. In a recent study published in November 2010 in *Vaccine*, Tebbens et al estimated the cost benefits from the adoption of GPEI to be $ 40-50 billion from 2008-2035. Most of these benefits are attributed to the prevention of the high hospitalization rates among children with paralytic poliomyelitis and subsequent disability prevention, improved productivity and reduced costs of rehabilitation. Low-income countries receive almost 85% of the estimated total net benefits, given the huge number of paralytic poliomyelitis prevented in their otherwise highly susceptible populations [23].

## Conclusions

AFP surveillance remains the gold standard method of poliomyelitis detection. This has increased people’s and clinicians’ awareness of the importance to promptly notify suspected cases and the timely transportation of stool specimens to the National Poliovirus Laboratory in Baghdad, or alternatively have more than one laboratory for poliovirus detection in the country, all of which are very useful measures to increase the surveillance performance in the country. Capture-recapture method can be used in order to get a more accurate estimate of the incidence of AFP in at least some parts of the country.

## Authors’ original submitted files for images

Below are the links to the authors’ original submitted files for images. Authors’ original file for figure 1 Authors’ original file for figure 2 Authors’ original file for figure 3 Authors’ original file for figure 4 Authors’ original file for figure 5 Authors’ original file for figure 6 Authors’ original file for figure 7
