# Supplementary figures and images for: An epidemiological analysis of acute flaccid paralysis and its surveillance system in Iraq, 1997-2011
Source: BMC Infect Dis. 2014 Aug 20;14:448. doi: 10.1186/1471-2334-14-448 (PMC4159501; doi:10.1186/1471-2334-14-448)

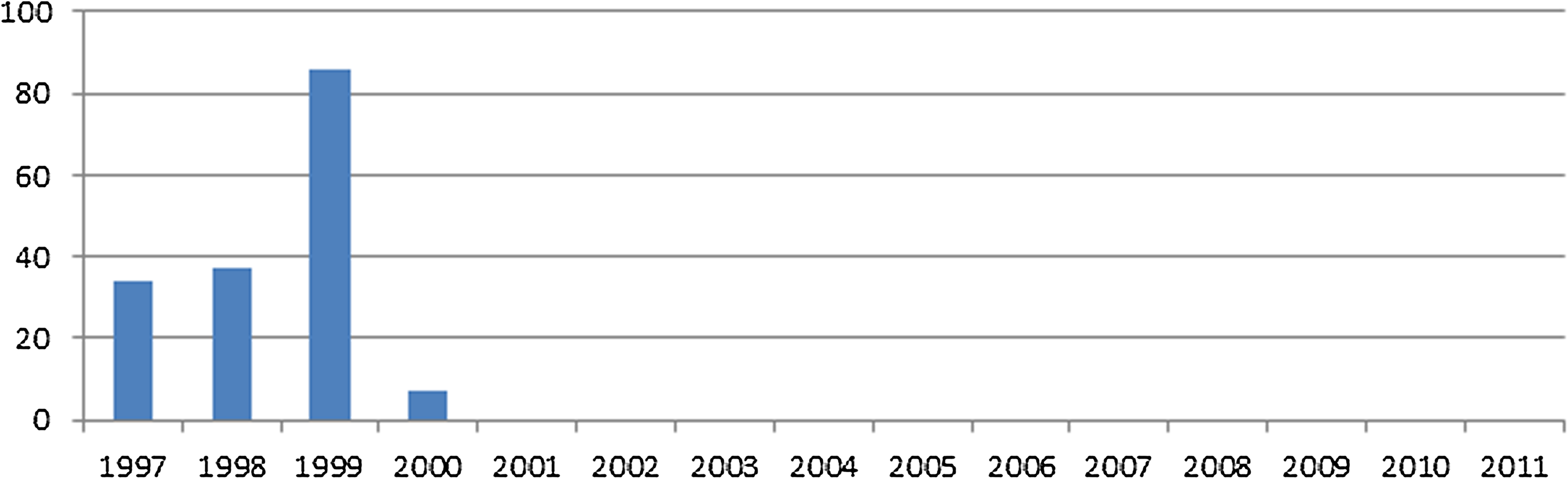

Supplement: Supplementary file 1 — Authors’ original file for figure 1 [file 12879_2013_3768_MOESM1_ESM.tiff]

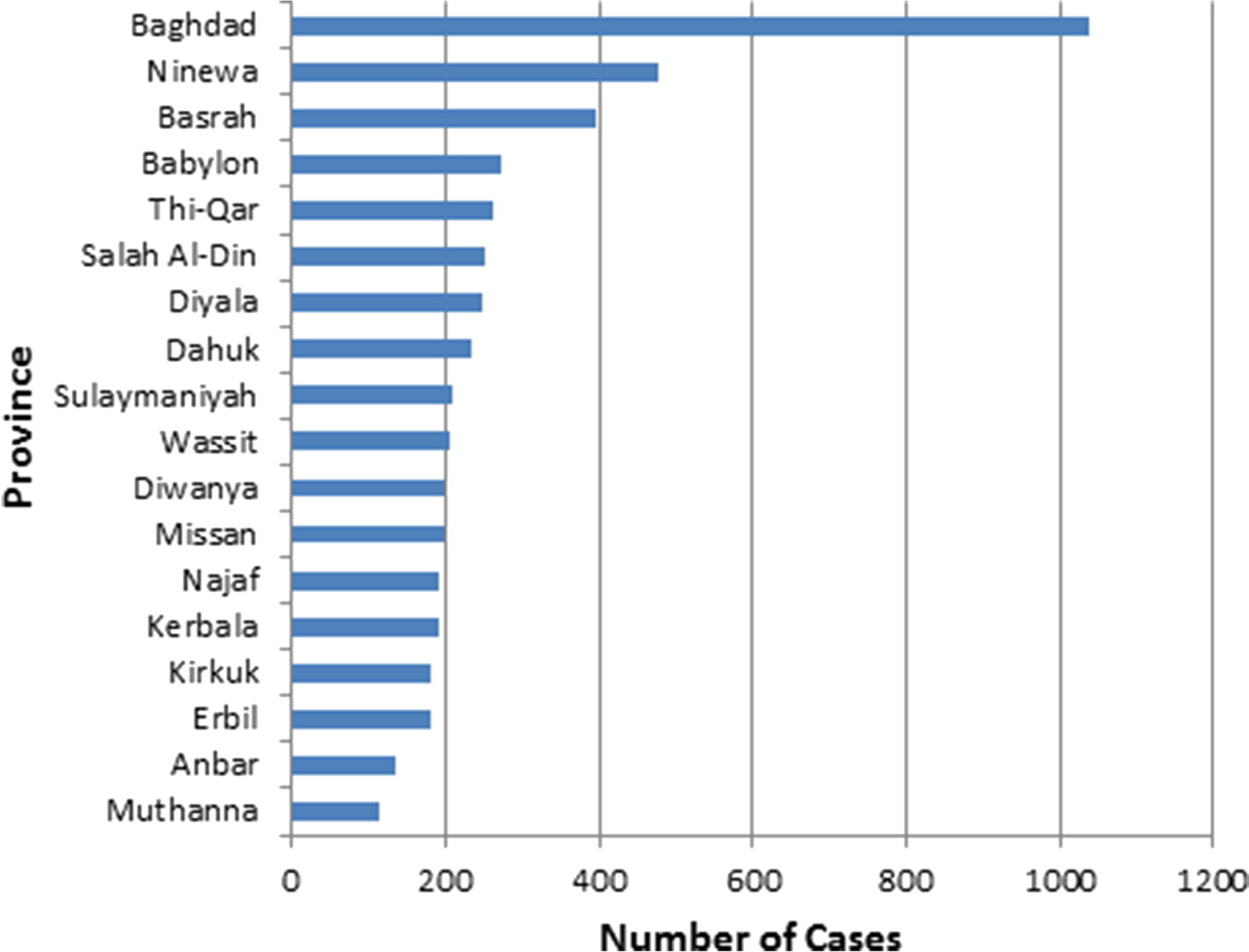

Supplement: Supplementary file 2 — Authors’ original file for figure 2 [file 12879_2013_3768_MOESM2_ESM.tiff]

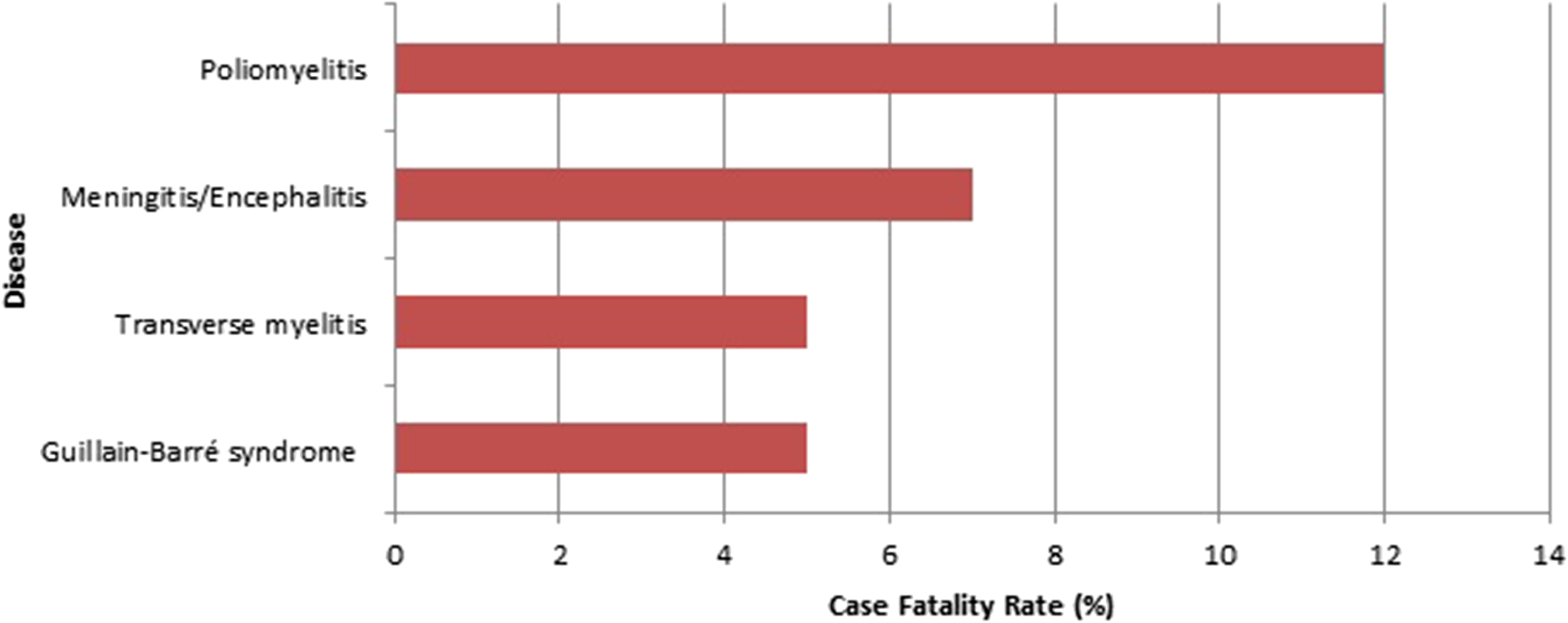

Supplement: Supplementary file 3 — Authors’ original file for figure 3 [file 12879_2013_3768_MOESM3_ESM.tiff]

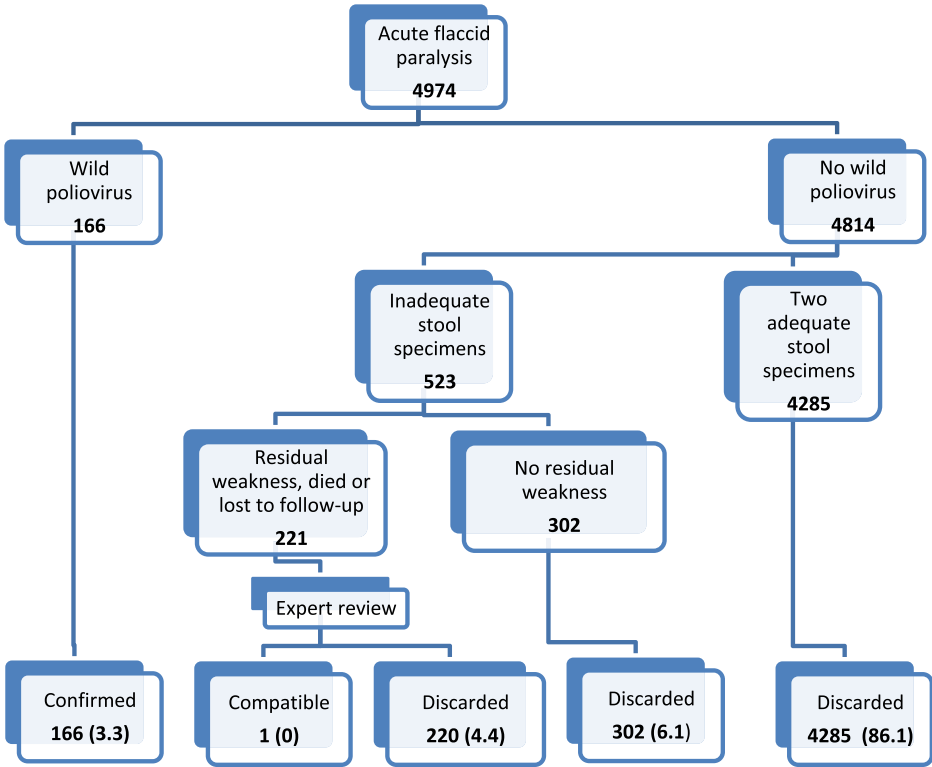

Supplement: Supplementary file 4 — Authors’ original file for figure 4 [file 12879_2013_3768_MOESM4_ESM.pdf]

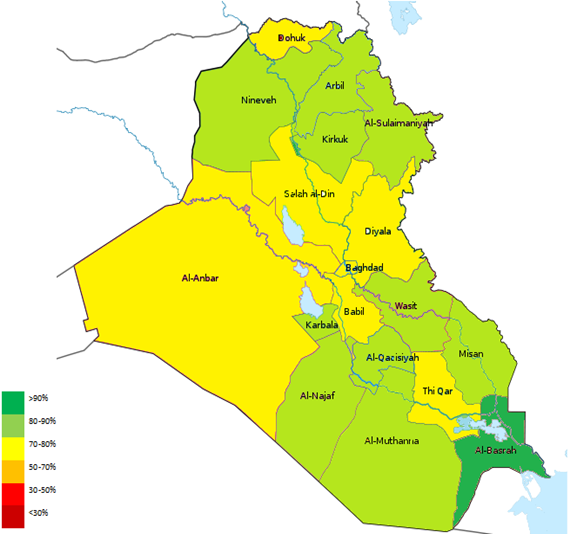

Supplement: Supplementary file 6 — Authors’ original file for figure 6 [file 12879_2013_3768_MOESM6_ESM.png]

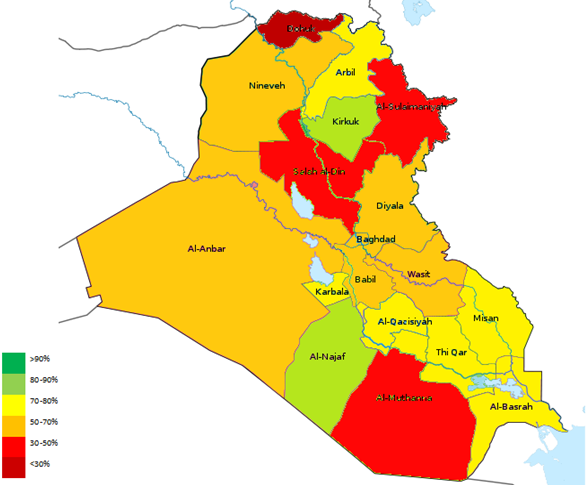

Supplement: Supplementary file 7 — Authors’ original file for figure 7 [file 12879_2013_3768_MOESM7_ESM.png]
