# Supplementary material for: An epidemiological analysis of acute flaccid paralysis and its surveillance system in Iraq, 1997-2011
Source: BMC Infect Dis. 2014 Aug 20;14:448. doi: 10.1186/1471-2334-14-448 (PMC4159501; doi:10.1186/1471-2334-14-448)
Supplement: Supplementary file 5 — Authors’ original file for figure 5 [file 12879_2013_3768_MOESM5_ESM.pdf]

Indicator	Target	Attribute	Actual performance															Average
			1997	1998	1999	2000	2001	2002	2003	2004	2005	2006	2007	2008	2009	2010	2011	
**No. of non-polio AFP cases per 100 000 population aged <15 years**	=>1	Sensitivity	1.1	1.1	1.7	2.5	2.6	2.8	1.8	2.3	2.5	3.0	3.2	3.3	2.9	3.4	3.5	**2.5**
**% of surveillance site providing routine report (including ' zero reports') on time**	=>80	Completeness of reporting	N/A	N/A	N/A	N/A	N/A	N/A	N/A	N/A	N/A	N/A	N/A	91	96	97	99	**96**
**% of AFP cases notified within 7 days from the onset**	=>80	Timeliness of notification	73	70	71	74	79	87	85	82	83	81	82	86	82	77	79	**79**
**% of AFP cases Investigated**	=>80	Completeness of investigation	100	100	100	100	100	100	100	100	100	100	100	100	100	100	100	**100**
**% of AFP cases investigated <48 hours**	=>80	Timeliness of investigation	85	89	90	89	93	95	90	87	88	90	87	95	97	95	98	**91**
**% of AFP cases followed up at least 60 days from the onset**	=>80	Completeness of follow -up	53	87	90	96	95	95	98	100	100	100	100	90	100	100	100	**94**
**% of AFP cases with two adequate stool specimens**	=>80	Timeliness and quality of stool collection	63	58	80	81	91	95	91	91	94	92	90	89	90	87	90	**85**
**% of stool specimens in the laboratory within 3 days of collection**	=>80	Timeliness	-	-	-	-	-	77	69	58	58	51	54	52	66	70	72	**63***
**% of specimen results sent from national laboratory within 28 days of receipt of the specimen in the laboratory**	=>80	Timeliness of reporting	-	-	-	-	-	98	30	66	99	93	99	100	100	100	100	**89**
**Stool specimens from which a non-polio enterovirus is isolated**	=>10	Quality of laboratory performance	10	10	18	20	23	12	18	27	25	20	12	10	27	18	19	18
